# Process Parameters Analysis in Diamond Wire Saw Cutting NdFeB Magnet

**DOI:** 10.3390/ma18051173

**Published:** 2025-03-06

**Authors:** Chengwanli Peng, Guanzheng Li, Xingchun Zhang, Yufei Gao

**Affiliations:** 1 SDU-ANU Joint Science College, Shandong University, Weihai 264209, China; 202200700123@mail.sdu.edu.cn; 2 Key Laboratory of High Efficiency and Clean Mechanical Manufacture of MOE, School of Mechanical Engineering, Shandong University, Jinan 250061, China; 3 State Key Laboratory of Advanced Equipment and Technology for Metal Forming, Shandong University, Jinan 250061, China; 4 Shandong Key Laboratory of High Performance Tools and System, Shandong University, Jinan 250061, China

**Keywords:** NdFeB magnet, diamond wire saw, surface waviness, orthogonal experiment, process parameters

## Abstract

Neodymium iron boron (NdFeB) magnetic materials are widely used in fields such as electronics, medical devices, power machinery, and hardware machinery. This paper conducted a three-factor and five-level orthogonal experiment on diamond wire saw cutting NdFeB to determine the influence degree of key factors such as workpiece feed rate, diamond wire speed, and workpiece processed size on the surface roughness *R*a and waviness *W*a of NdFeB slices. Further analysis was conducted on the influence of various parameters on the PV value (peak valley difference) of the waviness profile curve of the sawed surface. Finally, slicing processing was carried out under optimized process parameter combinations. The research results indicate that the primary and secondary order of process parameters affecting surface roughness *R*a and waviness *W*a is workpiece feed rate, wire speed, and sawed workpiece size, and the influence on the waviness PV value also shows a consistent trend. The optimal combination of processing parameters is workpiece feed rate of 0.1 mm·min^−1^, wire speed of 1600 m·min^−1^, and workpiece size of 10 mm. The obtained surface roughness *R*a is 0.433 μm and the waviness *W*a is 0.037 μm, respectively. The regression mathematical model for the waviness PV value is PV = 0.747 × *v*_s_^−0.342^ × *v_w_*^0.546^ × *L*^0.109^. The research results of this paper provide an experimental basis and guidance for optimizing process parameters of sawing NdFeB.

## 1. Introduction

**Figure 1:**
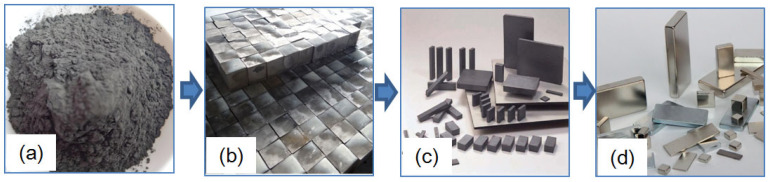
Different processing steps for sintered NdFeB magnet, (**a**) powdered raw materials, (**b**) sintered raw blocks, (**c**) sliced, (**d**) electroplated finished products.

Neodymium iron boron (NdFeB) magnetic materials are widely used in fields such as electronics, medical devices, power machinery, and hardware machinery. In recent years, NdFeB plays a more important role in new energy, wind power generation, communication technology, aerospace, and other fields, and its demand is constantly increasing [1,2]. The production process of NdFeB magnets mainly includes sintering powdered raw materials into blank blocks, and then cutting them into slices, which are then surface ground and electroplated to produce the final product. Figure 1 shows the several typical preparation stages of NdFeB magnets. The final process of NdFeB magnets is electroplating, which mainly makes their surface smoother and more beautiful, less prone to oxidation and corrosion, and extends their service life. Before electroplating, NdFeB slices generally need to be ground to improve surface quality. Therefore, the surface characteristics formed in the NdFeB slicing process directly affect the workload and cost of subsequent grinding processing.

Wire electrical discharge machining (WEDM) is the earliest method used for slicing NdFeB, and scholars’ research mainly involves temperature field analysis of NdFeB magnetism in WEDM, material removal mechanism, and the influence of WEDM process parameters [3,4,5,6]. These studies have found that WEDM-cut NdFeB magnet surfaces exhibit defects such as cracks, craters, and melting. Due to thermal effects during the cutting process, oxidation occurs on the cut surface, leading to an 18.9% increase in oxygen content. Some neodymium and iron elements exist in the form of oxides, such as FeO and Nd_2_O_3_ [3]. The depth of the heat-affected zone can reach up to 35 μm [4]. The minimum roughness *R*a is about 2 μm [5,6]. Laser cutting is another cutting NdFeB process. Wang et al. [7] studied the effect of laser cutting on the internal structure and properties of sintered NdFeB, and found that the structure in the laser cutting area is damaged due to the high-temperature effect of the laser beam. The cut area presents obvious laser cutting textures, and its surface roughness is six–seven times that of wire saw cutting. Ren et al. [8] numerically simulated the laser cutting temperature distribution of NdFeB using the finite element method, the research results found that the instantaneous temperature in the cut area can exceed 1500 °C. Ma et al. [9] found that the laser-cut NdFeB surface roughness exceeded 10 μm. Laser processing has the advantages of small cutting kerf and fast cutting speed, but due to the poor thermal resistance of NdFeB, it is easy to cause more surface cracks and larger surface roughness, and the cutting can lead to a 19.8% increase in oxygen content on the surface [3]. In addition, the cost of laser processing is high, so it has not been widely used in processing of NdFeB. Wet abrasive waterjet (WAWJ) cutting can effectively avoid the influence of cutting thermal damage, but the surface roughness is generally higher than 8 μm [3].

In recent years, the diamond wire saw cutting technology, which has the advantages of low cutting loss, high processing efficiency, environmental friendliness, and relatively low cost [10,11,12,13], has become the main cutting process of NdFeB. This process removes materials by diamond abrasives fixed on a steel wire surface. In fact, diamond wire saw technology has been widely used in cutting as mono-Si [11,14], poly-Si [15], sapphire [16,17], ceramics [18,19], and composite materials [20,21,22]. Moreover, scholars have conducted extensive research on the application of diamond cutting technology in these fields. Relatively, research on NdFeB magnet cutting is still insufficient. Liu et al. [23] conducted the diamond wire sawing experiment of NdFeB magnets, and found that the surface roughness *R*a can reach 0.6 μm. Wu et al. [24] designed cutting experiments and analyzed the influence of different process parameters on the surface quality of NdFeB. However, the parameter values selected in the above sawing experiments are relatively low, which differs significantly in actual industrial production. Our research group conducted a single-factor experimental analysis of wire saw cutting NdFeB by using process parameters values in industrial production. The experimental results showed that the surface roughness was mostly within the range of 1~3 μm [25]. It is necessary to reduce the surface roughness of slices by optimizing process parameters. Costa et al. [26] conducted an NdFeB scratching test, and found that as the scratching depth increased, the material removal underwent a transition from ductile to brittle.

Scholars have conducted many studies on the influence of diamond wire saw cutting process parameters on the sawed surface characteristics, which mainly focus on the sawed surface morphology characteristics and surface roughness. The concerned process parameters mainly include saw wire speed and feed rate, as well as parameters such as diamond wire tension and abrasive size. Sopori et al. [27] found that wire speed and feed rate have a significant impact on the characteristics of the cut surface. Gupta et al. [28] found that high wire speed reduces the surface roughness and subsurface damage depth of slices. Costa et al. [29] and Wang et al. [30] obtained similar conclusions for sawing mono-Si. Guo et al. [31,32] believe that increasing the wire speed and reducing the workpiece feed rate are beneficial for improving the wire bow and cutting force during sawing, thereby improving the cut surface quality. The above studies have found that the wire saw cut surface roughness is less than 2 μm. Yin et al. [33] and Liedke et al. [34] also found that when sawing workpieces with different sizes, the surface morphology and roughness also vary. Workpieces with different sizes result in different material removal rates under the same set of process parameters. In addition, when the workpiece size changes, lubrication, cooling, chip removal, and other issues during the cutting process will also change, which will affect the material removal characteristics. Therefore, the processed NdFeB size should also be considered as one of the factors.

Additionally, the surface saw marks in diamond wire sawing have attracted the attention of scholars. The saw marks present a periodic waviness along the workpiece feed direction [35], which has a significant impact on the processed surface quality and increases the cost of subsequent processing steps. In the study of sawing silicon wafers, it was found that the stress concentration caused by periodic waviness can reduce the mechanical properties of the wafer, thereby increasing the probability of wafer fracture along the saw mark [36]. In addition, the periodic waviness has a greater impact on slice warpage [37]. Therefore, reducing the waviness has positive significance for improving wafer quality and reducing the workload of subsequent processes.

Early research by Teomet [38] suggested that periodic waviness on the sawed surface were caused by random lateral forces generated by abrasives on the diamond wire surface during sawing. The lateral forces caused the diamond wire to oscillate laterally in the saw kerf, and an increase in feed rate resulted in higher peak-valley values. Lai et al. [35] found that the periodic saw marks on the sawed surface of sapphire crystals were related to the reciprocating operation of diamond wire. This discovery was also proposed in experiments of cutting NdFeB magnets [23,24,25]. Liu et al. [23] and Wu et al. [24] found the PV value (peak-valley difference) of the waviness profile curve of the sawed surface is around 1.5~8 μm. However, the parameter values used are generally low, such as a maximum wire speed about 100 m·min^−1^. Qiu et al. [11] believed that periodic saw marks were affected by the wire bow and wire lateral swing. The reciprocating moving of diamond wire can cause fluctuations in the material removal behavior on both sides, resulting in periodic changes in the cut width and causing saw marks. Regarding the improvement of saw marks, Li et al. [39] conducted research on a new diamond wire cutting process. However, there is currently little research on optimizing process parameters for improving saw marks.

Overall, there is a lack of systematic experimental research on the optimization of sawing process parameter combinations in diamond wire saw slicing NdFeB, which is of great significance for improving the surface quality and reducing the subsequent processing cost. This paper conducted a three-factor and five-level orthogonal experiment on diamond wire saw cutting NdFeB by using the parameter values in industrial production. The impact of key factors, such as workpiece feed rate, saw wire speed, and workpiece size, on the surface roughness *R*a and waviness *W*a of slices was determined through range analysis of the experimental results. Further analysis was conducted on the influence of various parameters on the PV value of waviness profile curve of sawed surface. Finally, the optimized process parameter combination was determined, providing experimental basis and guidance for sawing NdFeB.

## 2. Materials and Methods

### 2.1. Experimental Materials and Equipment

**Figure 2:**
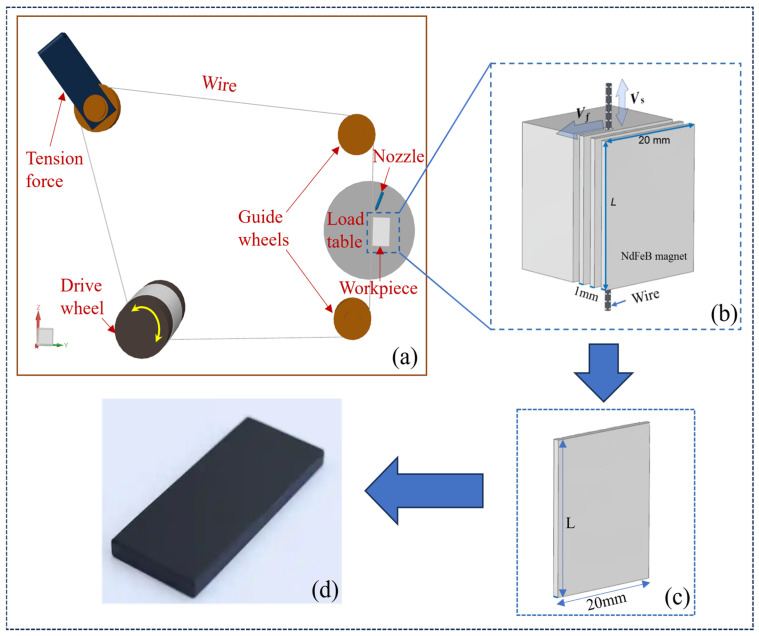
Experimental flow diagram. (**a**) Schematic of the wire saw machine; (**b**) magnified view of the machining process; (**c**) schematic of the slice dimensions;(**d**) NdFeB slices.

The wire sawing experiments were conducted on a diamond single-wire cutting machine (SH300, Guangzhou Shenghai Electronic Technology Co., Ltd., Guangzhou, China), and its appearance can be seen in our previously published paper [25]. A schematic diagram of the machine tool is shown in Figure 2a. The diamond wire is guided by two guide pulleys, and then wound onto the drive roller, with tension applied by a tension pulley using gravity. During the cutting process, the wire wound on the drive roller moves reciprocally at a set speed *V*_s_, while the load table feeds at a predetermined speed *V*_w_ to drive the NdFeB to press the wire. During sawing, tap water was used as the cutting fluid, and the nozzle sprays tap water onto the cutting area to achieve cooling and lubrication.

N35-sintered NdFeB rectangular magnets (provided by Ningbo Xinlin Magnetic Industry Co., Ltd., Ningbo, China) were adopted in the experiment, with a cut surface dimension of *L* × 20 mm, where *L* represents the workpiece dimension parallel to the wire direction and is investigated as a variable to explore its impact on the cutting quality. The dimension of the workpiece parallel to the feed direction was set to a constant 20 mm, and the slice thickness was set to 1 mm. A schematic of the slice dimensions is shown in Figure 2b. The parameters of the diamond wire (provided by Shantian New Materials Co., Ltd., Linyi City, China) used are listed in Table 1.

### 2.2. Experimental Design

In the experiment, the feed rate (*V*_w_), wire speed (*V*_s_) and workpiece size (*L*) were selected as three factors to investigate the effects of different parameters on the surface characteristics of NdFeB slices and obtain the optimal parameter combination. Values of process parameters are taken within the scope of industrial production, and each factor was assigned five levels and a three-factor. Then, a three factor and five-level orthogonal experiment was conducted. The sawed workpiece size (*L*) was adjusted by changing the dimension parallel to the wire moving direction. The experimental factors, levels, and parameter combinations involved in the orthogonal experiment are detailed in Table 2 and Table 3. Minitab Statistical Software version 2020 (State College, PA, USA) was used for analyzing the ranges and mean levels of various influencing factors on *R*a, *W*a, and PV values.

### 2.3. Evaluation Methods for Slice Surface Characteristics

**Figure 3:**
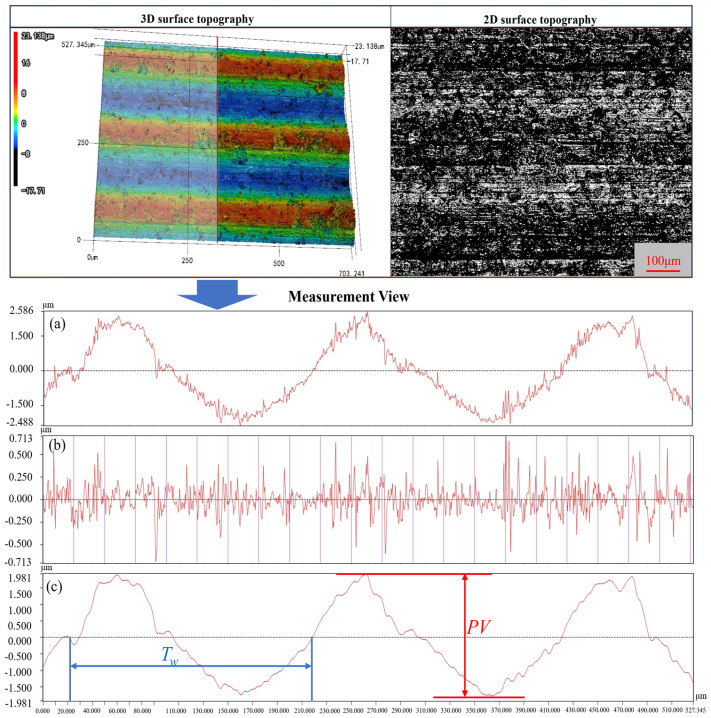
Three-dimensional and two-dimensional surface topography of the slice and its data measurement diagram (**a**) cross-section profile curve, (**b**) surface roughness profile curve, and (**c**) waviness profile curve.

After the wire sawing process, each group of slices was cleaned using an ultrasonic cleaner, and the surface morphology was observed using a laser confocal microscope VK-X200K (Keyence Corporation, Osaka, Japan). The surface roughness *R*a, waviness *W*a, and PV value of the slices were selected to evaluate the slice surface quality. For each slice, five measurement points were selected in the middle stable sawing area, and the average value was taken as the measurement result. As shown in Figure 3, the PV value represents the difference between the peak and valley of the waviness profile curve, and *T*_w_ denotes the waviness period. The arithmetic mean deviation of the waviness profile *W*a is calculated as follows [40]:

(1) $$W_{a}=\frac{1}{l_{m}}\int_{0}^{l_{m}}\left|Z_{\omega }\left(x\right)-Z_{m}\right|dx$$

where *l*_m_ is the sampling length for evaluating the waviness profile, *Z*_m_ is the average surface height, and $Z_{\omega }\left(x\right)$ is the profile curve function along the *x*-direction.

In the observational study previously published by our research group on the surface topography characteristics of NdFeB cut by a diamond wire saw [25], the sawed surfaces exhibit periodic waviness characteristics with alternating peaks and valleys. The sawed surface is created by pits caused by material brittle removal and scratches resulting from material ductile removal. These features together constitute the typical surface morphology of NdFeB processed by diamond wire sawing. The three-dimensional topography shown in Figure 3 shows the variation in the waviness period *T_w_* and the difference in the height of the peaks and valleys (PV) of the sliced surface, and the brittle craters on the sawed surface can be visualized in 3D topography (in dark blue) and 2D topography (in black).

It is noteworthy that previous study has found different processing parameter combinations have a significant impact on surface topography characteristics [23,24,25]. For example, they have a significant effect on the alternating frequency of peaks and valleys (periodic features) on the sawed surface. Additionally, the height difference between peaks and valleys varies with changes in processing parameters. The distribution of ductile scratches and brittle pits on the sawed surface also shows significant differences. These phenomena indicate that adjusting processing parameters has a crucial influence on the surface characteristic parameters such as *R*a, *W*a, and PV values.

## 3. Results and Discussion

### 3.1. Effect of Process Parameters on Surface Roughness

Table 4 presents the average surface roughness values of NdFeB slices under 25 different parameter combinations. Compared with the sawing experiment results of Liu et al. [23], when using the same workpiece feed rate, the surface roughness value obtained in our sawing experiment decreased. The main reason is that the increase in wire speed leads to an increase in the number of abrasives entering the cutting zone per unit time.

**Figure 4:**
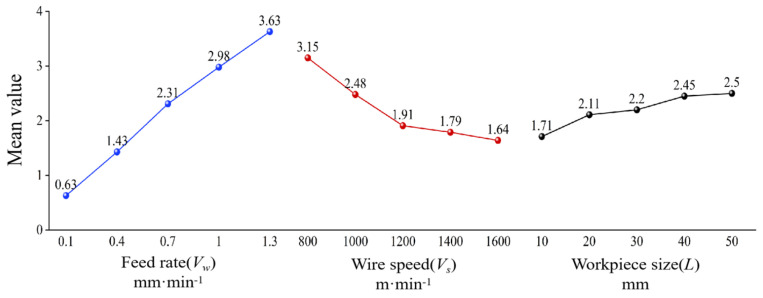
Mean levels of various influencing factors on roughness *R*a.

Table 5 presents the range analysis results of surface roughness *R*a. It can be observed that the range *R* values for feed rate, wire speed, and sawed workpiece size are 3.00, 1.51, and 0.79, respectively. This indicates that the feed rate has the greatest influence on surface roughness *R*a, while the workpiece size has the least influence. Therefore, the order of influence on surface roughness *R*a is feed rate (*V*_w_) > wire speed (*V*_s_) > sawed workpiece size (*L*). Figure 4 shows the mean values of the factors affecting surface roughness *R*a. It can be seen more intuitively that the decrease in feed rate and workpiece size, as well as the increase in wire speed, are beneficial for improving surface roughness.

From the range analysis results of the orthogonal experiment, it is evident that the feed rate has a significant impact on the surface roughness *R*a. Specifically, a higher feed rate results in a larger *R*a value, and the minimum *R*a value is achieved when the feed rate is 0.1 mm·min^−1^. The wire speed also influences *R*a value, with an increase in wire speed leading to a reduction in *R*a value. The minimum *Ra* value is observed at a wire speed of 1600 m·min^−1^. The workpiece size has the least effect on *R*a, and a smaller workpiece size corresponds to a lower *R*a value. The minimum *R*a value is obtained when the sawed workpiece size is 10 mm. Based on the above analysis, the optimal experimental parameter combination for minimizing surface roughness *R*a is *A*_1_*B*_5_*C*_1_. At this time, the workpiece feed rate is 0.1 mm·min^−1^, the wire speed is 1600 m·min^−1^, and the workpiece size is 10 mm.

### 3.2. Effect of Process Parameters on Surface Waviness

The surface waviness *W*a values of NdFeB slices under 25 groups of parameters were measured, as shown in Table 6. And Table 7 shows the range analysis results of the waviness *W*a. The range *R* values of the workpiece feed rate, saw wire speed, and sawed workpiece size on the surface waviness *W*a are 0.73, 0.2 and 0.12, respectively. It can be seen that the workpiece feed rate has the greatest impact on *W*a, while the sawed workpiece size has the smallest impact. The order of the impact on *W*a is as follows: workpiece feed rate (*V*_w_) > wire speed (*V*_s_) > workpiece size (*L*).

**Figure 5:**
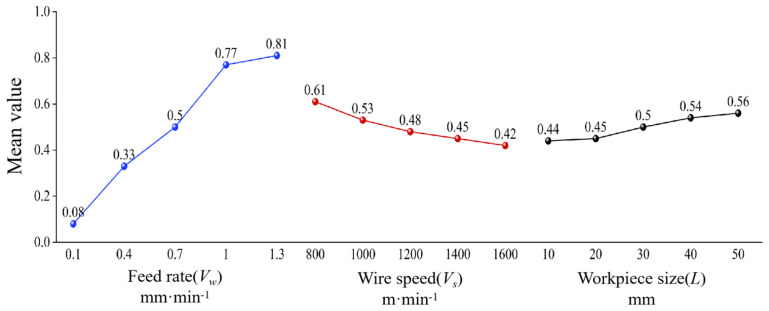
Mean levels of various influencing factors on waviness *W*a.

As shown in Figure 5, the average levels of various influencing factors on the waviness can be visually observed, which is that the decrease in feed rate and workpiece size, as well as the increase in wire speed, are beneficial for improving surface waviness. It can be concluded that the optimal process parameter combination for obtaining minimum surface waviness *W*a is *A*_1_*B*_5_*C*_1_. Correspondingly, the workpiece feed rate is 0.1 mm·min^−1^, the sawing wire speed is 1600 m·min^−1^, and the sawed workpiece size is 10 mm.

The existence of periodic waviness on the processed surface significantly reduces the flatness and surface quality of the slices. In the previous content, the variation law of waviness *W*a with processing parameters was explained. Here, further quantitative analysis is carried out by observing the peak valley difference (i.e., PV value) of the waviness contour curve to clarify the influence of feed rate, wire speed, and sawed workpiece size on PV value. The PV values of the sliced surfaces under various parameter combinations in the orthogonal experiment are shown in Table 8. Compared with the sawing experiment results of Liu et al. [23] and Wu et al. [24], the PV values of the sliced surface obtained in our sawing experiment decreased. The main reason for the analysis may be that the precision of the high-wire speed cutting equipment we use is higher than that of the low-wire speed sawing machine.

**Figure 6:**
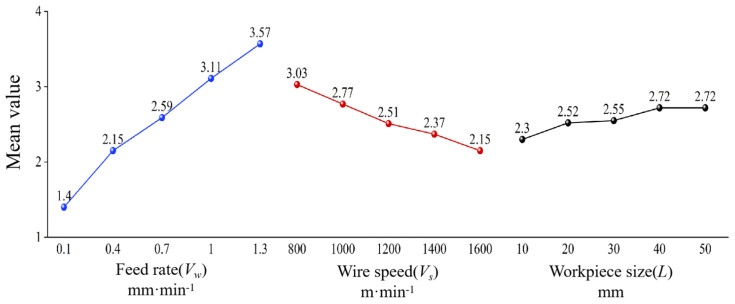
Mean levels of various influencing factors on PV value.

Table 9 shows the analysis results of the range of PV values on the sawed surface of NdFeB. According to the data in Table 9, the range *R* values of PV values for workpiece feed rate, wire speed, and workpiece size are 14.2, 8.7, and 4.22, respectively. It can be seen that the influence of workpiece feed rate on PV value is the greatest, while the influence of sawed workpiece size is the smallest. The order of the influence on PV value is obtained as workpiece feed rate (*V*_w_) > wire speed (*V*_s_) > sawed workpiece size (*L*). Based on the experimental analysis results shown in Table 9, it can be concluded that the optimal combination of experimental levels for obtaining minimum PV value is *A*_1_*B*_5_*C*_1_, which is consistent with the optimal experimental level derived from the analysis of *W*a. Correspondingly, the workpiece feed rate is 0.1 mm·min^−1^, the saw wire speed is 1600 m·min^−1^, and the sawed workpiece size is 10 mm. Figure 6 shows the average levels of various influencing factors on PV value. The analysis results indicate that the decrease in feed rate and workpiece size, as well as the increase in wire speed are beneficial for decreasing the PV values.

**Figure 7:**
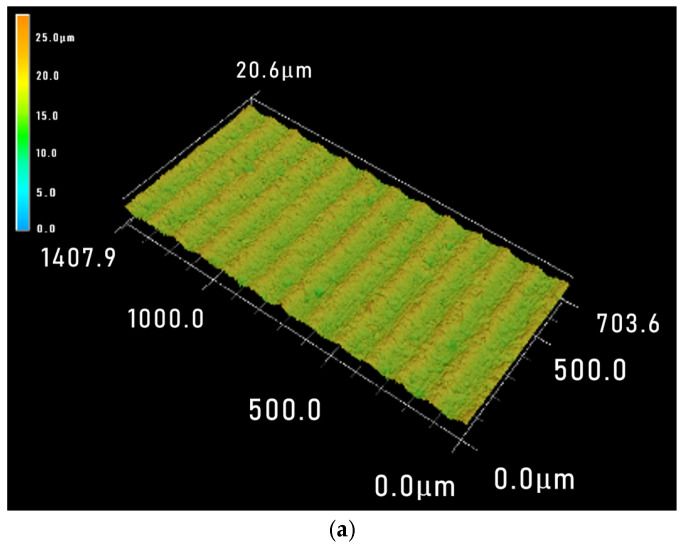

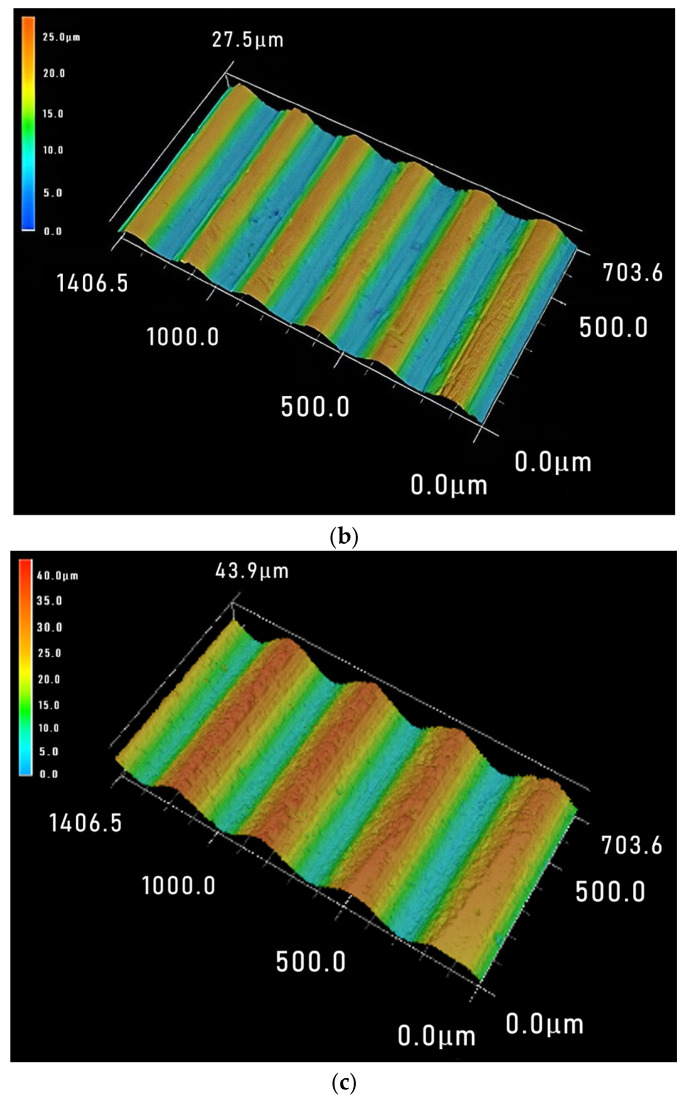
Three sets of slice 3D morphology under the condition of wire speed = 1200 m·min^−1^ (*V*_w_-*L*). (**a**) 0.4 mm·min^−1^-40 mm; (**b**) 1.0 mm·min^−1^-20 mm; (**c**) 1.3 mm·min^−1^-10 mm.

In order to more intuitively show the periodic changes in surface waviness, a larger range of three-dimensional images of sliced surfaces was observed. Figure 7 shows the sawed 3D surface image when using a wire speed of 1200 m·min^−1^, and the periodic waviness can be clearly seen. It can be observed that the sawed workpiece size has almost no effect on the waviness period *T_w_* of the sawed surface, while the larger the feed rate, the larger the period value *T_w_*, which is consistent with the research conclusion of Liu et al. [23]. For the PV value, although the sawed workpiece size decreases sequentially from Figure 7a–c, the increase in feed rate has a greater impact on the PV value, so the waviness PV values show an increasing trend from Figure 7a–c.

**Figure 8:**
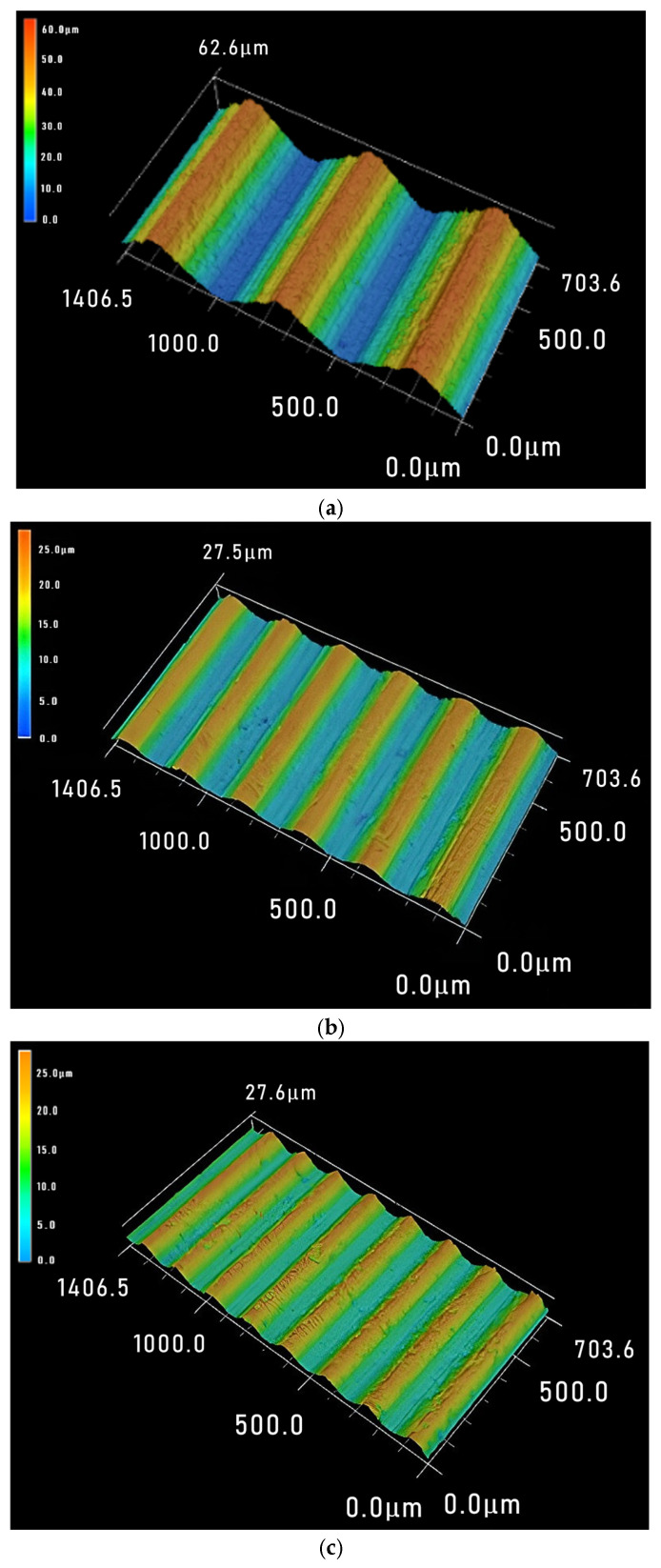
Three sets of slice 3D morphology under the condition of feed rate *v_w_* = 1.0 mm·min^−1^ (*V*_s_-*L*). (**a**) 800 m·min^−1^n-30 mm; (**b**) 1200 m·min^−1^-20 mm; (**c**) 1600 m·min^−1^-10 mm.

Figure 8 shows the surface 3D morphology under three different process parameters with a feed rate of 1.0 mm·min^−1^. From Figure 8a–c, the workpiece size decreases continuously, and the wire speed gradually increases. It can be seen that the PV value gradually decreases from Figure 8a–c, indicating that the effect will not conflict when both parameter values change simultaneously and have the same impact on the surface. For the waviness period *T_w_*, sawed workpiece size has almost no effect on it. And as the wire speed increases, the time it takes for the saw wire to run back and forth for one cycle decreases, so the feed distance decreases and the period *T_w_* decreases.

**Figure 9:**
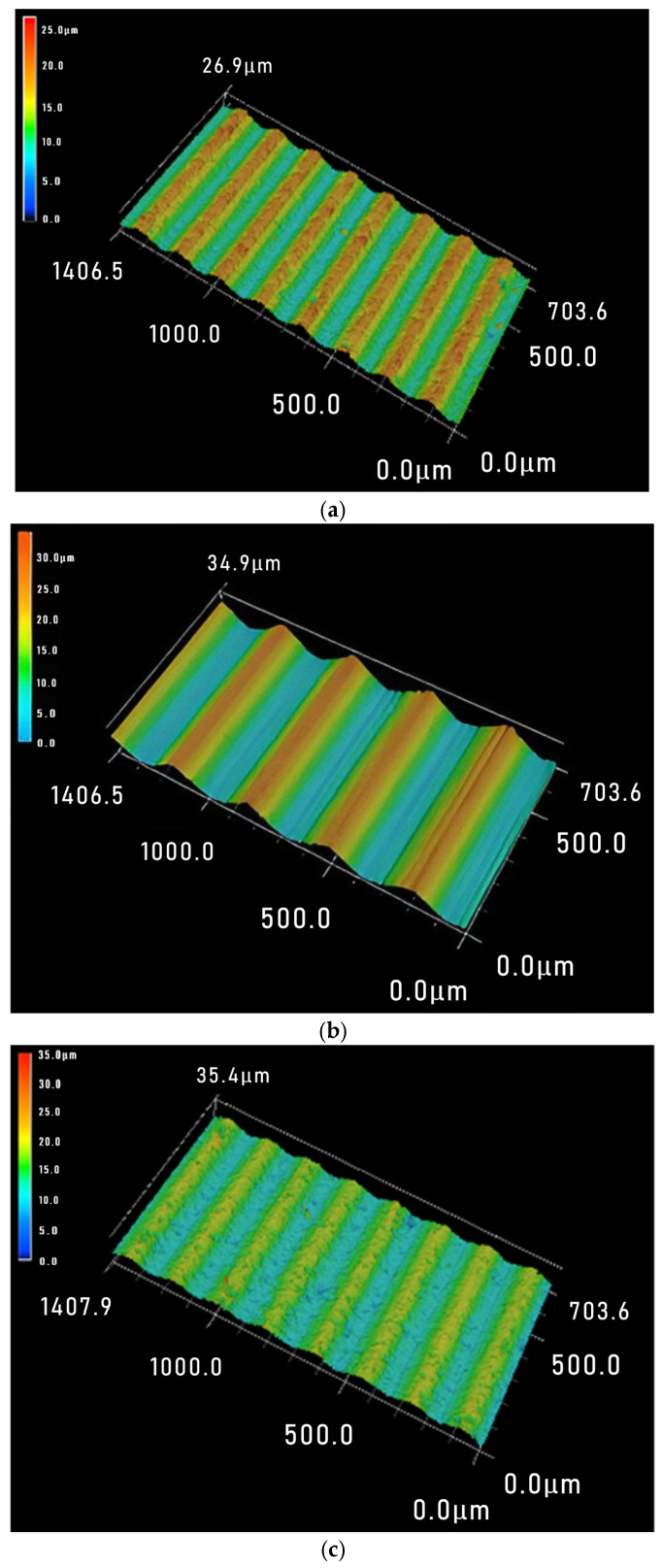
Three sets of slice 3D morphology under the condition of workpiece size *L* = 50 mm (*V*_w_-*V*_s_). (**a**) 0.4 mm·min^−1^-800 m·min^−1^; (**b**) 1.0 mm·min^−1^-1000 m·min^−1^; (**c**) 1.3 mm·min^−1^-1600 m·min^−1^.

Figure 9 shows the 3D surface morphology of three different process parameters with a sawed workpiece size of 50 mm. From Figure 9a–c, the feed rate and wire speed have both increased, and an increase in PV value can be observed. It can be seen that within the range of process parameters used in this paper, it is feasible to offset the negative impact of excessive feed rate on the sawed surface by increasing the wire speed. The waviness period *T_w_* is also influenced by the combined effect of feed rate and wire speed, which means it needs to be determined based on specific values.

The PV value of surface waviness has a more significant impact on subsequent grinding processes, so it is necessary to develop a regression equation for PV to achieve its prediction by establishing a mapping relationship between process parameters and PV values. In the orthogonal experimental design of this paper, the five level values of each of the three factors are equally spaced. Therefore, it is suitable to use polynomial regression analysis to obtain the regression equation. By conducting regression analysis on the processing parameters and PV values used in the orthogonal experiment of this paper, the mathematical theoretical model for PV values can be obtained by using the MATLAB Version 2020 (Natick, MA, USA) as follows:

PV = 0.747 × *V*_s_^−0.342^ × *V*_w_^0.546^ × *L*^0.109^ (2)

From the above Equation (2), it can be seen that when the feed rate and sawed workpiece size increase and the wire speed decreases, the PV value will increase. And the absolute value of the exponent of *V*_w_ is the largest, followed by *V*_s_, and *L* is the smallest. This indicates that feed rate has the most significant impact on PV value, while the sawed workpiece size has the least impact on PV value, which is consistent with the previous discussion.

### 3.3. Sawing Results by Using Optimal Process Parameter Combination

**Figure 10:**
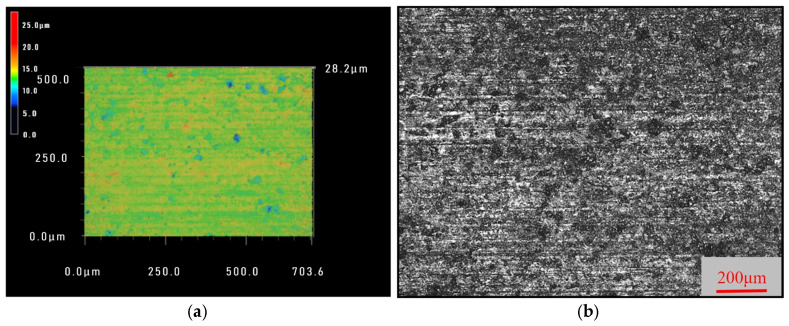
Three-dimensional (**a**) and two-dimensional (**b**) surface topographies under optimal processing parameter combinations: *V*_w_ = 0.1 m·min^−1^, *V*_s_ = 1600 m·min^−1^, and *L* = 10 mm.

The optimal process parameter combination predicted based on roughness *Ra* and waviness *W*_a_ is consistent, with a workpiece feed rate of 0.1 mm·min^−1^, a wire speed of 1600 m·min^−1^ and workpiece size of 10 mm. This parameter combination did not appear in the orthogonal experiment, so sawing experiments were conducted by using this parameter combination to verify the analysis correctness. The 3D and 2D images of the sawed surface morphology under this set of process parameters are shown in Figure 10. It can be seen that the processed surface is relatively smooth and flat. The roughness *R*a and waviness *W*a were measured at any five positions and their mean values were taken. The surface roughness *R*a was 0.433 μm and the waviness *W*a was 0.037 μm. The obtained *R*a and *W*a are both lower than the values under other processing parameters in the orthogonal experiment, which is consistent with the analysis results and proves the rationality of the conclusion.

## 4. Conclusions

This paper conducted a three-factor and five-level orthogonal experiment on diamond wire saw cutting NdFeB magnetic materials. The surface roughness *R*a and waviness *W*a were used as evaluation indicators to explore the influence of workpiece feed rate, wire speed and workpiece size on the sawed NdFeB surface characteristics. The PV value of the sawed surface waviness profile curve was further analyzed. The main conclusions obtained are as follows:

(1) The sawing process parameters show a consistent trend in their impact on surface roughness *R*a and waviness *W*a. The decrease in workpiece feed rate and size, as well as the increase in wire speed, are beneficial for improving surface roughness and waviness. The order of the influence of various processing parameters on surface roughness *R*a and waviness *W*a is workpiece feed rate, wire speed, and workpiece size, and the influence of process parameters on the waviness PV value also shows a consistent trend.
(2) The optimal combination of process parameters based on minimum *R*a and *W*a is consistent, which is that workpiece feed rate is 0.1 mm·min^−1^, wire speed is 1600 m·min^−1^, and workpiece size is 10 mm. Correspondingly, *R*a is 0.433 μm and *W*a is 0.037 μm. The regression equation for PV values established based on experimental data are PV = 0.747 × *V*_s_^−0.342^ × *V*_w_^0.546^ × *L*^0.109^.

The research results have clarified the order of the influence of process parameters on the quality of cutting NdFeB by diamond wire saw, and provided optimized parameter combinations, which provides an experimental basis and guidance for the sawing process. However, further exploration is needed to determine the impact of the interaction between multiple process parameters on the cut NdFeB surface properties during wire sawing, in order to achieve more precise optimization of process parameter combinations.

## Figures and Tables

**Table 1:** Parameters of diamond wire.

Parameters	Values
Diamond saw wire length (m)	70
Diameter of saw wire core (μm)	220
Type of abrasives	Nickel-coated diamond
Abrasive size (μm)	70–85
Abrasive distribution density (grits·mm^−1^)	15–20
Tensile limit of saw wire (MPa)	3275
Moh’s hardness of saw wire (nickel plating)	5

**Table 2:** Factors and levels of the orthogonal experiment.

Levels	Factors		
	(A) Feed Rate (*V*_w_) /mm·min^−1^	(B) Wire Speed (*V*_s_) /m·min^−1^	(C) Workpiece Size (*L*) /mm
1	0.1 (A_1_)	800 (B_1_)	10 (C_1_)
2	0.4 (A_2_)	1000 (B_2_)	20 (C_2_)
3	0.7 (A_3_)	1200 (B_3_)	30 (C_3_)
4	1.0 (A_4_)	1400 (B_4_)	40 (C_4_)
5	1.3 (A_5_)	1600 (B_5_)	50 (C_5_)

**Table 3:** Experimental parameter combinations.

No.	Parameter Combinations			No.	Parameter Combinations		
1	$A_{1}$	$B_{1}$	$C_{1}$	14	$A_{3}$	$B_{4}$	$C_{5}$
2	$A_{1}$	$B_{2}$	$C_{3}$	15	$A_{3}$	$B_{5}$	$C_{2}$
3	$A_{1}$	$B_{3}$	$C_{5}$	16	$A_{4}$	$B_{1}$	$C_{3}$
4	$A_{1}$	$B_{4}$	$C_{2}$	17	$A_{4}$	$B_{2}$	$C_{5}$
5	$A_{1}$	$B_{5}$	$C_{4}$	18	$A_{4}$	$B_{3}$	$C_{2}$
6	$A_{2}$	$B_{1}$	$C_{5}$	19	$A_{4}$	$B_{4}$	$C_{4}$
7	$A_{2}$	$B_{2}$	$C_{2}$	20	$A_{4}$	$B_{5}$	$C_{1}$
8	$A_{2}$	$B_{3}$	$C_{4}$	21	$A_{5}$	$B_{1}$	$C_{2}$
9	$A_{1}$	$B_{4}$	$C_{1}$	22	$A_{5}$	$B_{2}$	$C_{4}$
10	$A_{2}$	$B_{5}$	$C_{3}$	23	$A_{5}$	$B_{3}$	$C_{1}$
11	$A_{3}$	$B_{1}$	$C_{4}$	24	$A_{5}$	$B_{4}$	$C_{3}$
12	$A_{3}$	$B_{2}$	$C_{1}$	25	$A_{5}$	$B_{5}$	$C_{5}$
13	$A_{3}$	$B_{3}$	$C_{3}$				

**Table 4:** Surface roughness *R*a values of NdFeB slices.

No.	*R*a (μm)	No.	*R*a (μm)	No.	*R*a (μm)
1	0.634	10	1.129	19	2.501
2	0.679	11	3.489	20	1.720
3	0.732	12	2.247	21	4.847
4	0.522	13	2.051	22	4.319
5	0.579	14	2.239	23	2.979
6	2.382	15	1.512	24	2.732
7	1.281	16	4.416	25	3.263
8	1.387	17	3.875		
9	0.964	18	2.378		

**Table 5:** Range analysis of surface roughness *R*a.

Parameters	Levels	*K* Values	*K*_avg_ Values	Optimal Level	Range *R* Value
*A*	$A_{1}$	3.15	0.63	$A_{1}$	3.00
	$A_{2}$	7.14	1.43		
	$A_{3}$	11.54	2.31		
	$A_{4}$	14.89	2.98		
	$A_{5}$	18.14	3.63		
*B*	$B_{1}$	15.77	3.15	$B_{5}$	1.51
	$B_{2}$	12.40	2.48		
	$B_{3}$	9.53	1.91		
	$B_{4}$	8.96	1.79		
	$B_{5}$	8.20	1.64		
*C*	$C_{1}$	8.54	1.71	$C_{1}$	0.79
	$C_{2}$	10.54	2.11		
	$C_{3}$	11.01	2.20		
	$C_{4}$	12.27	2.45		
	$C_{5}$	12.49	2.50		

**Table 6:** Surface waviness *W*a values of NdFeB slices.

No.	*W*a (μm)	No.	*W*a (μm)	No.	*W*a (μm)
1	0.083	10	0.244	19	0.709
2	0.085	11	0.713	20	0.645
3	0.086	12	0.489	21	0.866
4	0.069	13	0.497	22	0.879
5	0.074	14	0.475	23	0.756
6	0.513	15	0.327	24	0.767
7	0.303	16	0.897	25	0.803
8	0.346	17	0.913		
9	0.237	18	0.695		

**Table 7:** Range analysis of surface waviness *W*a.

Parameters	Levels	*K* Values	*K*_avg_ Values	Optimal Level	Range R Value
*A*	$A_{1}$	0.40	0.08	$A_{1}$	0.73
	$A_{2}$	1.64	0.33		
	$A_{3}$	2.50	0.50		
	$A_{4}$	3.86	0.77		
	$A_{5}$	4.07	0.81		
*B*	$B_{1}$	3.07	0.61	$B_{5}$	0.2
	$B_{2}$	2.67	0.53		
	$B_{3}$	2.38	0.48		
	$B_{4}$	2.26	0.45		
	$B_{5}$	2.09	0.42		
*C*	$C_{1}$	2.21	0.44	$C_{1}$	0.12
	$C_{2}$	2.26	0.45		
	$C_{3}$	2.49	0.50		
	$C_{4}$	2.72	0.54		
	$C_{5}$	2.79	0.56		

**Table 8:** PV values of NdFeB slices in orthogonal experiment.

No.	PV (μm)	No.	PV (μm)	No.	PV (μm)
1	1.415	10	1.789	19	2.916
2	1.425	11	1.802	20	2.317
3	1.432	12	3.271	21	4.024
4	1.345	13	2.631	22	3.885
5	1.375	14	2.553	23	3.373
6	2.785	15	2.005	24	3.315
7	2.217	16	3.633	25	3.243
8	2.149	17	3.673		
9	1.789	18	3.021		

**Table 9:** Range analysis of PV values.

Parameters	Levels	*K* Values	*K*_avg_ Values	Optimal Level	Range R Value
*A*	$A_{1}$	6.99	1.40	$A_{1}$	2.17
	$A_{2}$	10.74	2.15		
	$A_{3}$	12.94	2.59		
	$A_{4}$	15.56	3.11		
	$A_{5}$	17.84	3.57		
*B*	$B_{1}$	15.13	3.03	$B_{5}$	0.88
	$B_{2}$	13.83	2.77		
	$B_{3}$	12.53	2.51		
	$B_{4}$	11.84	2.37		
	$B_{5}$	10.74	2.15		
*C*	$C_{1}$	11.52	2.30	$C_{1}$	0.42
	$C_{2}$	12.61	2.52		
	$C_{3}$	12.73	2.55		
	$C_{4}$	13.60	2.72		
	$C_{5}$	13.61	2.72		

## Data Availability

The original contributions presented in this study are included in the article. Further inquiries can be directed to the corresponding author.
